# Development of predictive indices for evaluating the UHI adaptation potential of green roof- and wall-based scenarios in the Mediterranean climate

**DOI:** 10.1038/s41598-024-67567-9

**Published:** 2024-09-17

**Authors:** Tiziana Susca, Jacopo Iaria, Fabio Zanghirella

**Affiliations:** 1 ENEA Italian National Agency for New Technologies, Energy and Sustainable Economic Development, Rome, Italy; 2 Department of Biological, Geological, and Environmental Sciences (BiGeA), Alma Mater Studiorum University of Bologna, Bologna, Italy

**Keywords:** Climate sciences, Environmental sciences, Engineering

## Abstract

Urban heat islands can jeopardize urban inhabitants, but the installation of green roofs (GRs) and walls (GWs) can contribute to mitigating urban overheating. The present study provides novel indices to easily predict the spatial median variation in air temperature at pedestrian heights related to the application of GR- and GW-based scenarios during the hottest hours of a typical summer day by varying the building height (BH), coverage percentage, and leaf area index. The indices are meant to be applied to built areas with 0.3–0.4 urban density in the Mediterranean climate and are derived from regression models fed with the outputs of 281 simulations of three urban areas developed and run in ENVI-met software. The developed models are all highly significant. The GR model shows that mitigation is influenced by all three parameters, and it can estimate mitigation with a root mean square error of 0.05 °C. Compared with the other parameters, the GW models revealed that the BH did not influence the decrease in air temperature. The green façade and living wall (LW) indices predict mitigation with errors of 0.04 °C and 0.05 °C, respectively. However, for the LW model, further parameters should be considered to improve its reliability.

## Introduction

The urban heat island (UHI), an increase in urban temperature compared to that of rural areas^1^, is a serious issue globally^2^ that can jeopardize urban inhabitants’ health^3^, increase building cooling energy demand^4,5^, and contribute to air quality and ecosystem deterioration^6,7^. It is therefore vital to adapt cities to UHIs. Greenery offers an opportunity to reduce urban overheating^8,9^, and although residual urban spaces are difficult to find in densely urbanized cities, building façades and rooftops can easily accommodate vegetation. Building integrated vegetation technologies (BIVTs), namely, green roofs and walls, can concomitantly reduce UHIs and energy consumption for summer cooling^10–12^.

Green roofs (GRs) are characterized by a horizontal growing medium layer. Depending on the thickness of the substrate, green roofs are clustered into extensive green roofs (EGRs) when the thickness of the growing medium is < 12 cm^13^ and intensive green roofs (IGRs) when it is > 15 cm^14^. Because of their light weight, EGRs can be extensively installed on existing buildings without structural intervention^15^. In contrast, because of their heavy load, IGRs can be installed only on buildings specifically designed to support their weight^14^. Green walls (GWs) can be distinguished according to the position of the substrate: green façades (GFs) are characterized by a horizontal substrate while living walls (LWs) have a vertical substrate adherent to the wall^16^. Due to the beneficial effects of BIVT deployment, many city councils—for example, London^17^, Sydney, and Brisbane^18^—incentivize their installation. However, because of the economic investment related to the application of BIVT-based UHI adaptation plans^19^, their potential effectiveness should be assessed beforehand. Tools to forecast the UHI mitigation potential of BIVT plans do exist (e.g.,^20–22^), but they require specific knowledge, computational facilities, and time^23^, which are often missing.

The use of simpler tools, such as indices, to predict the effectiveness of the application of BIVTs might incentivize the development, early-stage evaluation, and application of UHI adaptation plans. To the best of our knowledge, little research has been carried out on the development of such predictive indices. For instance, using artificial neural networks, a study (i.e.,^24^) forecasts the surface UHI mitigation potential of green roofs limited to rooftop surfaces rather than at the pedestrian level. Although of great interest, the model outputs can hardly be used by urban planners to verify beforehand the effectiveness of adaptation plans. Moreover, Sinsel et al.^25^ used a regression model to assess the UHI mitigation potential of GRs, cool roofs, and super cool roofs depending only on the variation of building height. Some studies focused on the effect of land use cover and change on UHI (e.g.,^26–28^). For instance, Liu et al.^20^ investigated the surface UHI based on future land use simulations using the FLUS model, which is an integrated model for multi-type land use scenario simulations by coupling human and natural effects. Likewise, another study (i.e.,^29^) focused on developing an index to forecast UHI mitigation based on evapotranspiration from vegetation, cooling distance of large urban parks, and albedo. Both the previous studies omit the potential impact of GW and GR deployment. Another research focused on assessing the effect of GR installation on UHI adaptation in Turin^25^. The study is based on mapping Turin and correlating the land surface temperature values with some urban features, such as height-to-width street ratio and building density; however, the study is limited to the potential UHI mitigation effect of GR installation in Turin.

Another study (i.e.,^30^) provided a model for the estimation of air temperature variation due to the installation of GRs by linking evapotranspiration to mitigation. However, to include easier-to-apply predictors such as urban geometry and percentage of coverage, high computational effort is necessary, hindering the wide use of the developed model.

The present study aims to fill this scientific gap by providing indices capable of easily predicting the variation in spatial median temperature in the hottest hour of a typical summer day at the pedestrian level (i.e., 1.5 m above the ground) related to the application of adaptation scenarios based on the installation of a single BIVT. Specifically, we developed UHI adaptation scenarios based on the single application of GFs, LWs, and EGRs; while IGRs were omitted because of their limited installation potential in built areas. The equations are meant to be applied to urban areas with urban density, defined as the ratio between buildings' basal area and the total surface of the considered zone, ranging from 0.3 to 0.4. Urban density has also been addressed as a local climate zone (LCZ), expanding the concept from just surfaces to regions of uniform surface cover, material, human activity, and phenomenon^31^. Furthermore, since the background climate is influential on GR and GW performance^10,11^, the indices were calibrated for three study areas in three Italian cities in the Mediterranean climate zone since the latter is experiencing a great intensification of heat stress^32,33^. Specifically, the selected cities belong to the Csa climate zone according to the Köppen Geiger classification^34^.

The developed indices based on parametric equations calculate the variation in air temperature at the pedestrian level by varying the most influential parameters for BIVT performance in mitigating urban air temperature overheating^35^: building height (BH), coverage percentage (COP) (i.e., the percentage of the surface occupied by BIVTs) and leaf area index (LAI) of the installed plants. The equations were obtained through regression models populated by the median air temperature variation at 1.5 m for 250 mitigation scenarios from 31 control scenarios. All the scenarios were developed and simulated in ENVI-met software^36^.

## Methods

**Figure 1:**
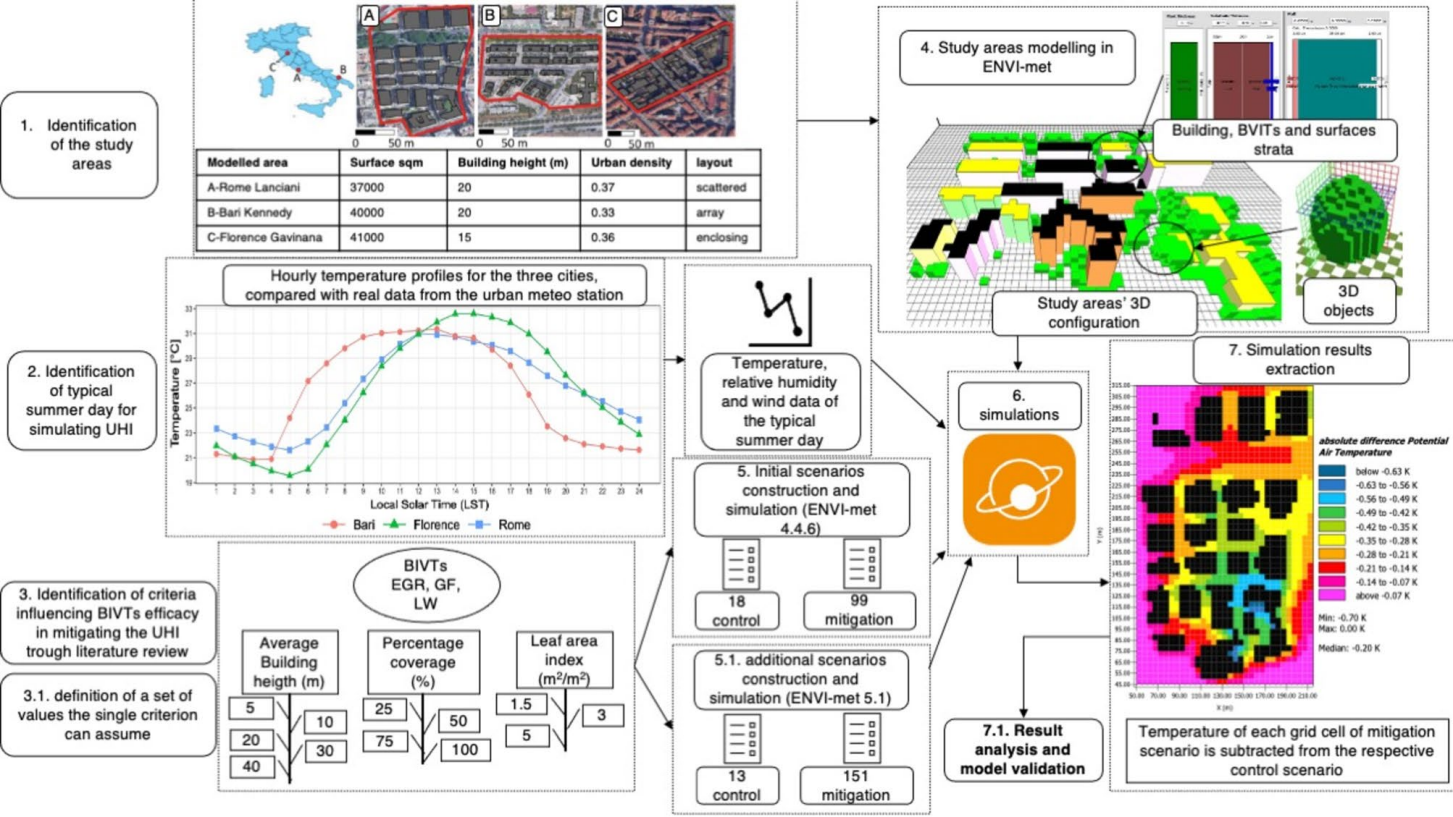
Methodology workflow.

For the development of the indices, three urban areas prone to UHI formation in Rome, Bari, and Florence were selected. The study areas of Via Lanciani in Rome, Viale Kennedy in Bari, and the neighborhood of Gavinana in Florence are characterized by similar extensions, LCZs, and urban densities (Fig. 1, 1. Identification of the study areas). The three urban areas were chosen to represent the main typical regional urban layouts^37,38^ to generalize the results as much as possible. Specifically, the Lanciani urban area presents a scattered layout with similar-sized squared buildings and roads arranged in the N‒S and E‒W directions; the selected urban area in Bari shows an array layout with buildings arranged along parallel streets, while Florence buildings are characterized by an enclosing layout with buildings forming closed polygons with internal courtyards. Another requisite for the selection of the areas was the access to monitored historical meteorological data recorded by a weather station positioned within the selected built areas and another station positioned in the rural environs. These latter data were used to force the ENVI-met models (Fig. 1). The methodology used has been thoroughly described in a previous study^35^; however, the present study included 164 new scenarios (Table S1).

All the models were run in ENVI-met for the typical summer day for each city. To identify the typical summer days, we compared the average hourly temperature profile of the hottest month (data from^39^) with the real-day temperature profile recorded at the urban stations after removing the rainy days and selecting the day with the smallest root mean square error (RMSE) and mean absolute error (MAE).

### UHI-mitigation criterion and value selection

Three parameters are predicted to influence UHI mitigation by BIVTs: BH^11,12,38,40^, COP^41^, and LAI^40^. For the development of mitigation and control scenarios, we assumed the following BHs: 5, 10, 20, 30, and 40 m. The incremental height was chosen as a multiple of the grid used for the development of the scenarios (see “ENVI-met simulations”). The maximum height was set at 40 m since previous research has shown that above this threshold, GRs have no mitigation potential^32^. For the COP, we considered 25, 50, 75, and 100%. GR coverage scenarios were modelled by applying GRs to 25%, 50%, and 75% of each building's roof surface throughout the study area, following the methodology proposed by Morakinyo et al.^41^ (Supplementary materials Fig. 1). Besides, for GWs coverage scenarios we first estimated buildings’ wall surface by multiplying the perimeter by the height, then individual buildings were randomly selected with the only constraint that the sum of their wall area would be 25, 50, and 75%, respectively (Supplementary materials Figs. 2, 3, 4). This method was preferred to applying GWs to single façades to avoid varying mitigation effects due to different façade orientations^40^.

Eventually, the LAI values were set to 1.5, 3, and 5 for low, medium, and high foliage density, respectively. All the mitigation scenarios were created by applying a single BIVT to the study area with combinations of the values defined for the three criteria.

### Modeling of the study areas

The selected areas were modeled in ENVI-met. The software requires the 3D arrangement of all urban elements (e.g., buildings, trees, shrubs, paved and natural surfaces, BIVTs) as well as their inner structure and physical properties to simulate the interaction between them and produce microclimatic outputs such as the potential air temperature. Additional information necessary to improve the model accuracy is meteorological data used as forcing conditions. Data from the urban weather stations were used to test the ENVI-met accuracy and to validate the baseline scenarios, while data from the rural weather stations were used to force the baseline and mitigation scenarios (see methodology in^35^).

### Scenarios

In addition to the 117 original scenarios developed by^35^, created by varying the value of one parameter per time while keeping the other constant at the maximum value, we added 164 additional intermediate scenarios—scenarios with the parameters changing together at low values—to account for BIVT efficacy under suboptimal conditions and to improve the results. The intermediate scenarios are representative of the suboptimal LAI (i.e., LAI 1.5) and coverage percentage conditions (i.e., COP 25%, 50%, and 75%) for the different BH classes in the three study areas.

Out of the 281 total scenarios, six are the baseline scenarios (i.e., three baseline scenarios developed by means of ENVI-met 4.4.6, and three using ENVI-met 5.1. See Methods—“ENVI-met simulations” section), namely those replicating the status quo of the urban areas; 25 are control scenarios, which are scenarios based on the status quo of the three urban areas and modified by varying the BH, according to what reported in Methods—“UHI-mitigation criteria criterion and value selection” section, without applying any BIVT; and 250 are adaptation scenarios, namely those based on control scenarios and developed and simulated by implementing a single BIVT each time and varying the COP and LAI singularly or concomitantly.

All the scenarios are shown in Table S.1 in the supplementary materials.

### ENVI-met simulations

Every scenario was simulated using the same grid parameters. Specifically, we choose a grid of 5 m (x) × 5 m (y) × 5 m (z) to obtain accurate outputs in a relatively short computational time ranging from six to eight hours. As suggested in the ENVI-met website, for each simulation, we added 10 cells to the border of the buildings to improve model stability; moreover, two days were simulated, the first one for stabilizing the model and the second one for obtaining the microclimate outputs^42,43^. All the simulations were run with an AMD Ryzen 53,600 6-Core processor and 32.0 Gb of RAM. The original scenarios were simulated using ENVI-met 4.4.6, while for the additional scenarios, ENVI-met 5.1 was used. The difference in the ENVI-met version required the baseline and the control scenarios to be simulated in both 4.4.6 and 5.1 to nullify any difference between the different releases.

### Data extraction and analysis

Using ENVI-met LEONARDO, we extracted the potential air temperature data at the pedestrian level related to the hottest hour in the three study areas in CSV format, namely, 1.00 p.m. for Rome and 2.00 p.m. for Bari and Florence, of all the simulated scenarios. By means of^44^, we calculated for each cell of the modeled areas the difference between the mitigation scenario and the control scenario (e.g., mitigation scenarios in Rome with a BH equal to 5 m were subtracted from the control scenario of Rome with the same BH value). Subsequently, to minimize the effect of inflows and outflows, the outputs of the simulated areas were cut one cell further from the perimeter of the most peripheric buildings, thus considering only the urban areas without the additional cells inserted for the simulation. Then, we calculated the mean, median, and 1^st^ and 3^rd^ quartile air temperature differences of each mitigation scenario. Subsequently, we used multiple linear regression models to calculate the mitigation index of each BIVT (functionlm(), baseline R). We tested heteroskedasticity with a Breusch‒Pagan test, package lmtest^45^ function bptest(), and normality with function shapiro.test() in baseline R; Cook’s distance was considered by plotting the model’s residuals. Eventually, we used the functions rmse() and mae() of^46^ to assess the RMSE and MAE of the equations in predicting mitigation. For the graph packages, ggplot2 and gghalves^47^ were used.

## Results

### Air temperature variation

**Figure 2:**
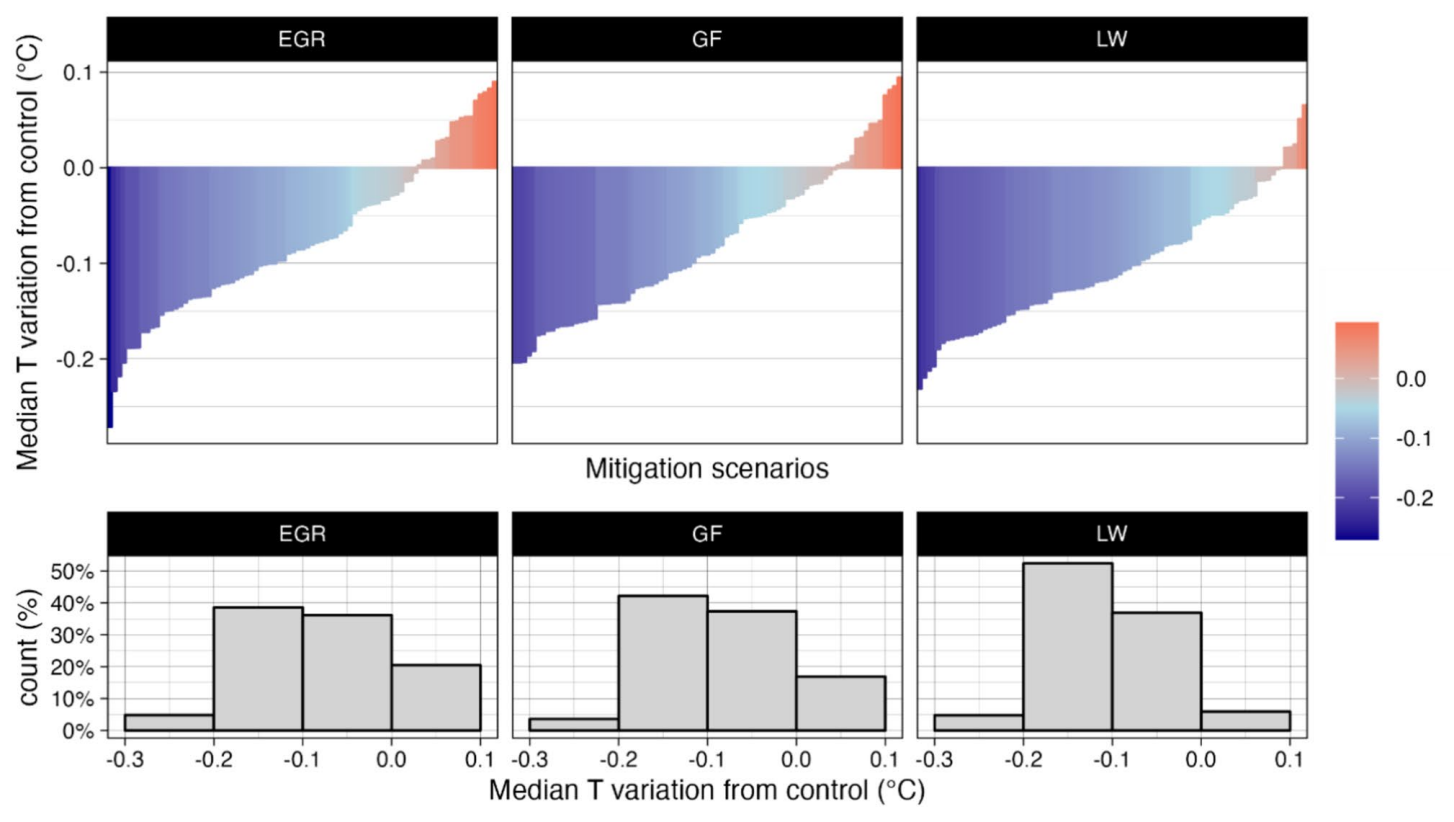
Median variation in air temperature of the mitigation scenarios grouped by BIVT.

Figure 2 shows the median difference in air temperature between the mitigation scenario and the control scenario for each investigated BIVT. Five percent of the EGR-based scenarios show a median mitigation effect greater than − 0.2 °C, 39% of the scenarios mitigate by − 0.2 to − 0.1 °C, 36% by − 0.1 to 0 °C, and 20% have a positive variation in temperature. The maximum mitigation is − 0.27 °C and is achieved by a scenario in Bari with an LAI of 5, COP of 100%, and BH of 5 m; the highest increase in temperature, + 0.08 °C, is found in Rome in a scenario with a 1.5 LAI, 25% COP and 20 m BH. Similarly, for GFs, only 4% of the scenarios achieve median mitigation equal to or greater than − 0.2 °C, 35%, and 39% of the scenarios show mitigation between − 0.2 and − 0.1 °C and between − 0.1 and 0 °C, respectively; in addition, 22% of the temperature variation induced by the GF installation is positive. The maximum mitigation of the GF reaches − 0.20 °C and is achieved in Florence when a scenario characterized by an LAI of 5 covering 100% of the 40 m high buildings is applied. The greatest increase in temperature, equal to + 0.09 °C, is found in Bari in a scenario with a GF LAI of 1.5 covering 25% of the 20 m high buildings. Eventually, 4% of the LW-based scenarios mitigate more than − 0.2 °C, 50%, and 39% of which have mitigation temperatures ranging from − 0.2 to − 0.1 °C and from − 0.1 to 0 °C, respectively. Only 6% of the LW scenarios produce a positive temperature variation. The highest decreases and increases in temperature are − 0.21 and + 0.07 °C, respectively. The former is achieved in Rome in a scenario with LAI 5, COP 100%, and BH 20 m and the latter is achieved in Bari with LAI 1.5, COP 25%, and BH 20 m.

For the baseline scenarios, RMSE, MAE, and the coefficient of determination have been calculated by comparing measured and simulated urban air temperatures. Such values have been compared with those found in the literature (i.e.,^48^) and assessing the accuracy of ENVI-met software in simulating the air temperature within the built environment, and it has been found that the air temperature outputs of the simulated areas are accurate enough.

### Indices

We used multiple linear regression models to calculate the relationships between the median air temperature variation and the BH, COP, and LAI to obtain three novel indices. Such indices are meant to predict the spatial median temperature variation at the pedestrian level when applying a specific BIVT. According to the user needs, the COP is expressed as an integer (i.e., for a 20% COP, 20 must be inserted), the mean BH in the built area is expressed in meters, and the LAI varies from a minimum value of 1.5 to a maximum value of 5.

For the EGRs, the equation is:

1 $${I}_{EGR}=-0.0784 -0.0016COP +0.0722\text{ln}\left(BH\right) -0.0177LAI$$

**Figure 3:**
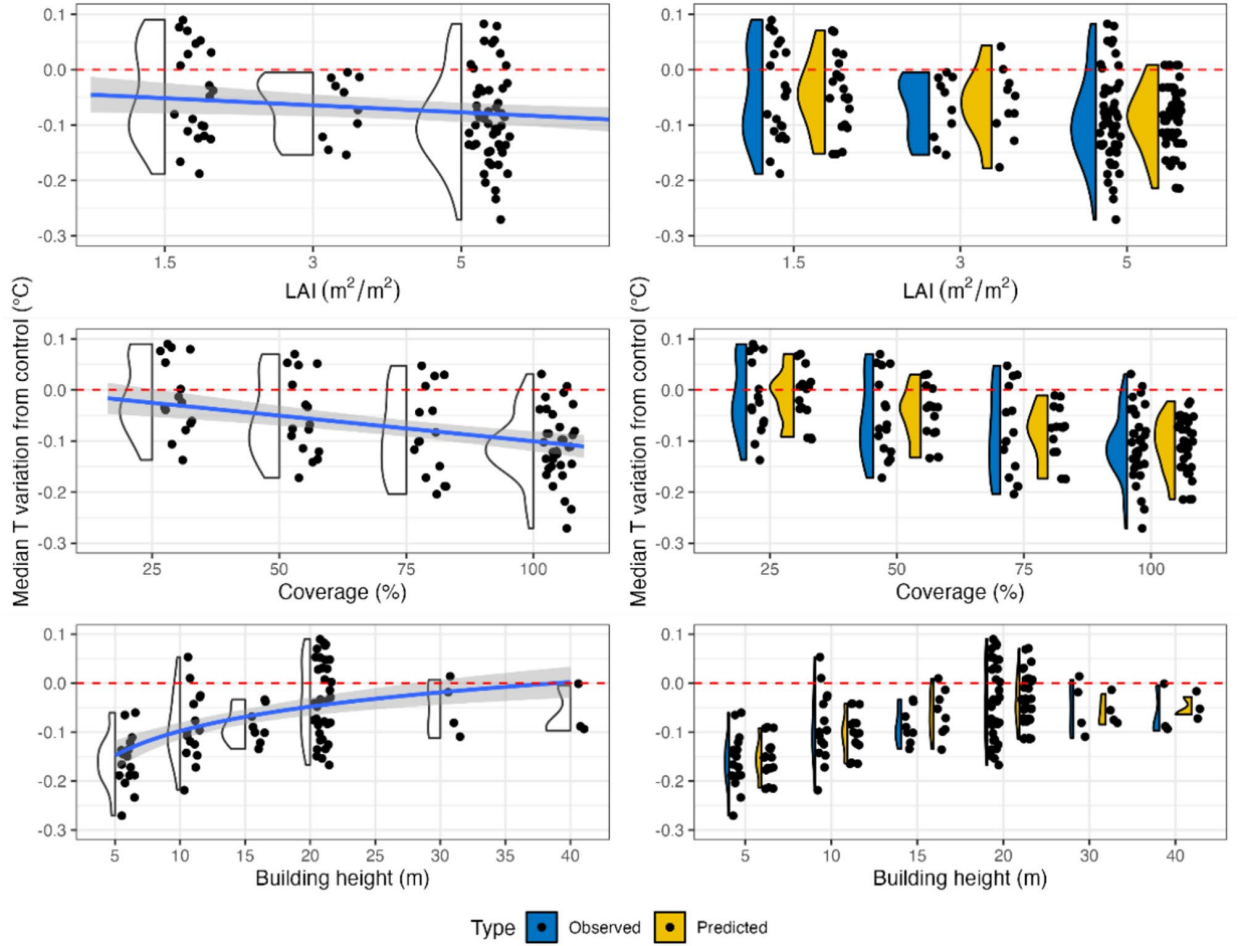
Right panel: comparison between observed and predicted values for the EGR model. Left panel: relationships between the median temperature variation and the predictors with trend lines for the EGR model.

**Figure 4:**
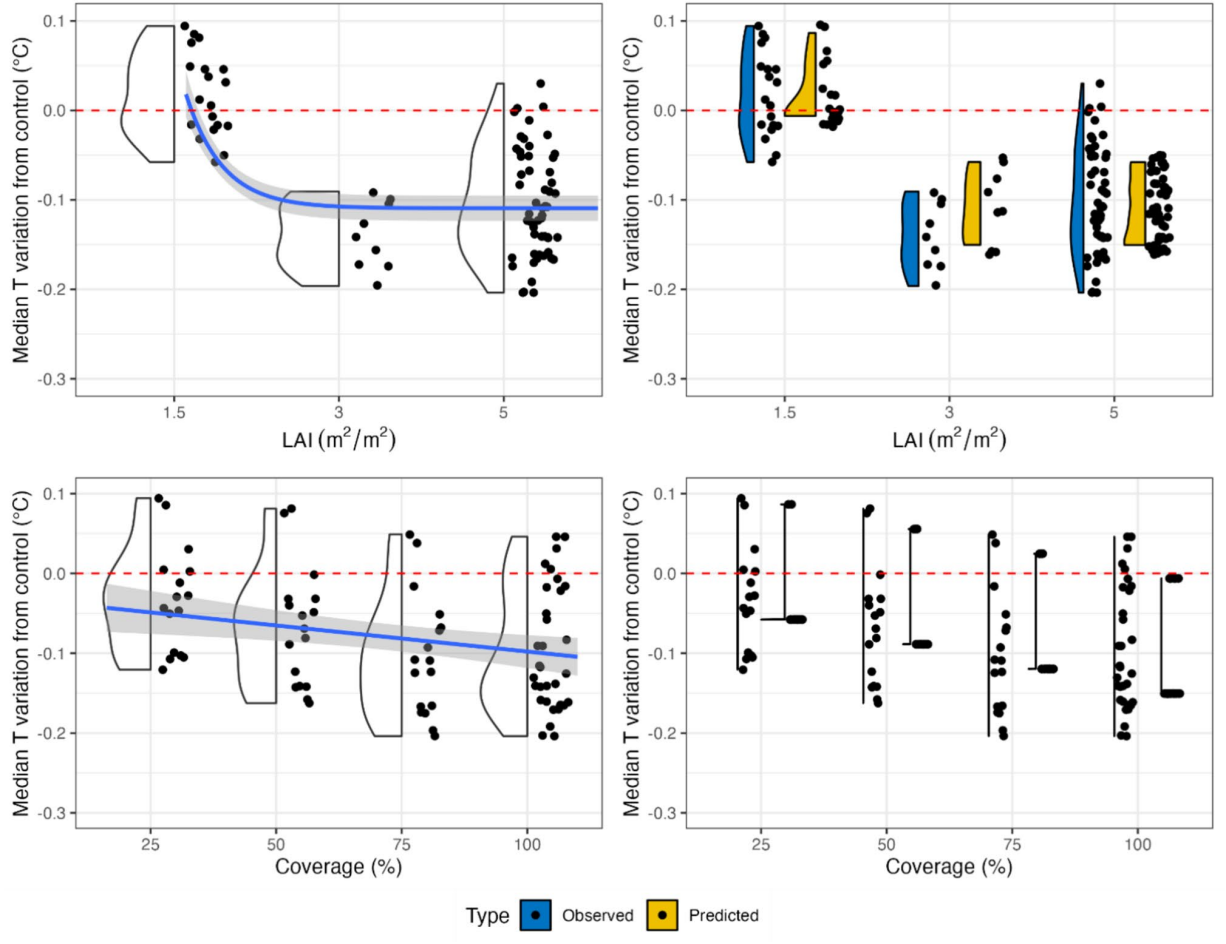
Right panel: comparison between observed and predicted values for the GF model. Left panel: Relationships between the median temperature variation and the predictors with trend lines for the GF model.

**Figure 5:**
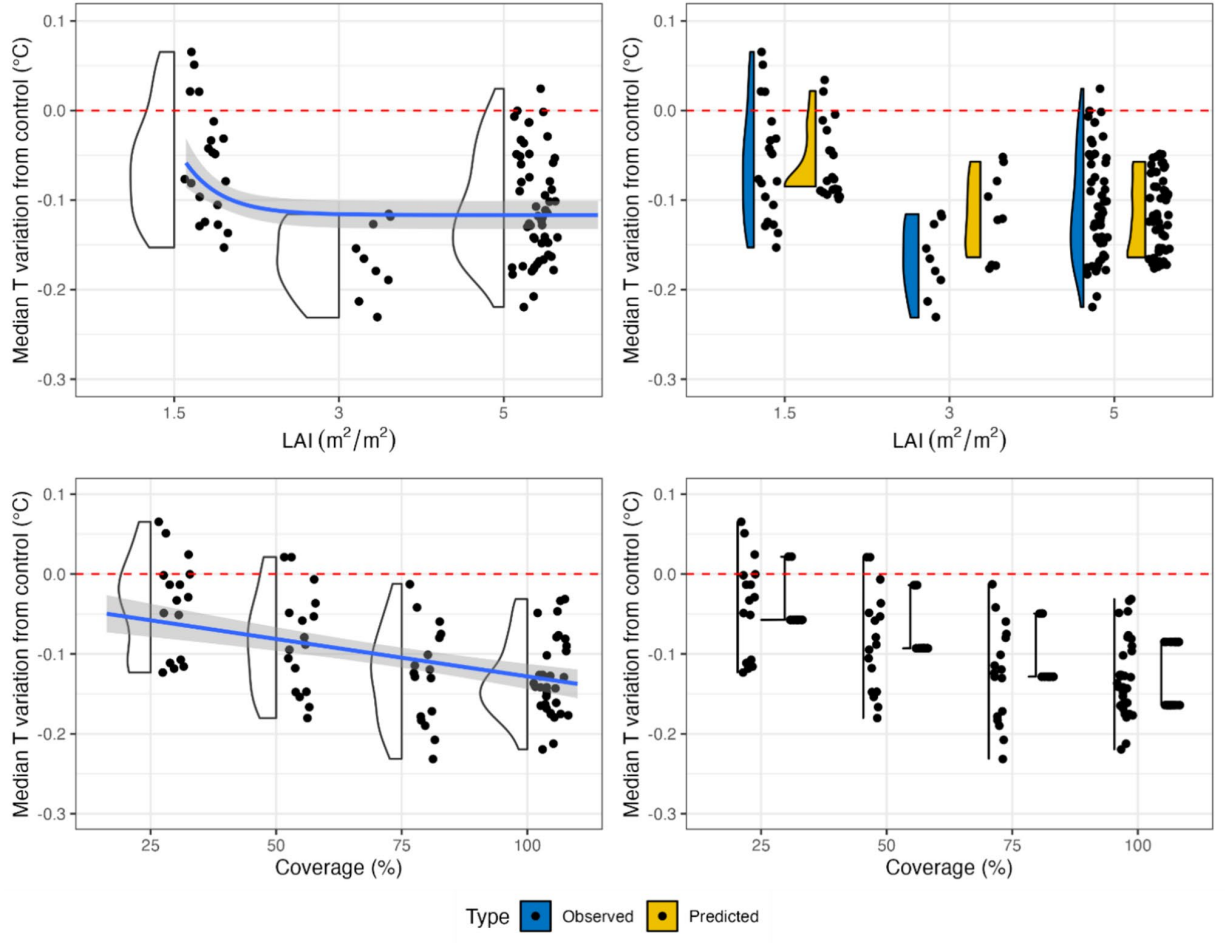
Right panel: comparison between the observed and predicted values for the LW model. Left panel: Relationships between the median temperature variation and the predictors with trend lines for the LW model.

Table 1 shows that the EGR model is significant and explains 61% of the variance in the data. Additionally, all the predictors are significant in the model, and their relationships with the temperature variation are depicted in Fig. 3. Specifically, Fig. 3, as well as Fig. 4 and Fig. 5, show in blue color the observed and in yellow the predicted temperature variations. The term “observed” refers to the difference in temperature between mitigation scenarios and control scenarios, while “predicted” temperature variations are referred to the values derived by the regression models. All the models were tested for normality of residuals using the Shapiro‒Wilkins test and for homoscedasticity using the Breusch‒Pagan test, as well as for the influence of single observations on the regression using Cook’s distance. For the EGRs, the residuals were normal (p-val = 0.06) and homoscedastic (p-val = 0.22). One observation was removed because it showed that Cook’s distance was greater than all the others (i.e., EGR with an LAI of 1.5 COP 25 BH 10 in Florence). As a measure of the errors in the indices, we computed the RMSE and MAE between the predicted and observed results and obtained values of 0.049 °C and 0.041 °C for the EGR index, respectively.

**Table 1:** Multiple linear regression of the EGR model outputs.

Extensive green roof				
Median T reduction = a + b*COP + c*ln(BH) + d*LAI				

Model coefficients	N. obs	Adj. R^2^	F value	p value
	82	0.61	43.45	10^–16^

Variables coefficients	Estimate	St. Err	t value	p value
a	− 0.0784	0.0353	− 2.22	0.03
b	− 0.0016	0.0002	− 8.11	10^–12^
c	0.0722	0.0095	7.58	10^–11^
d	− 0.0177	0.0038	− 4.63	10^–6^

For the GFs, we derived the following index:

2 $${I}_{GF}= -0.0269 -0.0012COP +123{.3e}^{-4.5\cdot LAI}$$

Table 2 shows the GF model coefficients’ estimates, standard errors t, and p values. The model neglects BH since it has been found to be nonsignificant. GF’s model explains 68% of the variance and has a p value equal to 10^–16^. The residuals are normal (p-val = 0.07) and homoscedastic (p-val = 0.07). One observation (i.e., GF LAI 1.5 COP 25 BH 10 in Florence) was removed since it was an outlier. Figure 4 depicts the relation between the single predictor and the median air temperature variation in the scenarios. The results show that predictions occupy a smaller range than the observed values and that they assume higher values when the LAI is equal to 3. The estimated RMSE and MAE for the index were 0.043 °C and 0.036 °C, respectively.

**Table 2:** Multiple linear regression of the GF model outputs.

Green façade				
Median T reduction = a + b*COP + c*exp(−4.5*LAI)				

Model coefficients	N. obs	Adj. R^2^	F value	p value
	83	0.68	88.82	10^–16^

Variables coefficients	Estimate	St. Err	t value	p value
a	− 0.0269	0.0126	− 2.134	0.03
b	− 0.0012	0.0002	− 7.270	10^–10^
c	123.3	9.947	− 12.39	10^–16^

For the LWs, we obtained the following index:

3 $${I}_{LW }=-0.0014COP + 67.55{e}^{-4.5\cdot LAI}$$

As for the GFs, BH was also a nonsignificant predictor for LWs and was removed to improve the model’s residuals. Similarly, the intercept (i.e., *a* value) was found not significantly different from zero and, therefore, it has been removed from Eq. 3. The model is significant and has an adjusted R^2^ of 0.50 (Table 3), and the residuals are homoscedastic (p-val = 0.1) but nonnormal (p-val = 0.007). One observation (i.e., LAI 1.5 COP 25 BH 10 in Florence) presented a Cook’s distance of magnitude greater than that of the other observations and was removed. The predicted values for LWs occupy a smaller range of values than the observed values and tend to cluster (Fig. 5), as well as underestimating the reduction for LAI 3; the RMSE and MAE for the indices are equal to 0.045 °C and 0.038 °C, respectively.

**Table 3:** Multiple linear regression of the LW model outputs.

Living wall				
Median T reduction = a + b*COP + c*exp(-4.5L*AI*)				

Model coefficients	N. obs	Adj. R^2^	F value	p value
	83	0.50	42.31	10^–13^

Variables coefficients	Estimate	St. Err	t value	p value
a	-0.022	0.0134	-1.614	0.11
b	-0.001423	0.0002	-7.813	10^–11^
c	67.55	10.51	6.426	10^–9^

## Discussion

This study provides a tool to ease the beforehand assessment of UHI adaptation plans based on scenarios developed by applying single BIVTs. Specifically, the authors developed three equations capable of providing median UHI mitigation values in an urban area by varying the COP, BH, and LAI of EGRs, GFs, or LWs. Compared to previous research, the main aim of the present study is to provide an easy-to-use tool that can be widely used by city council technicians and urban planners since it requires a limited amount of input data and computational time.

To the best of our knowledge, only a few studies focused on the interaction between BIVTs and pedestrian-level air temperature exist and are based on complex physical models. For example, Alexandri et al.^49^ developed an ad hoc dynamic microscale model that analyzes the effect of GRs and GWs on the air temperature in urban canyons and at the rooftop level; Djeddjig et al.^50^ developed a TRNSYS hygrothermal model of GWs and a model of mass flows in street canyons. Both studies provide an estimation of air temperature variation within an urban canyon rather than in a selected urban area. Therefore, they cannot be easily and widely used for UHI mitigation forecasting since they provide punctual air temperature values and require many parameters and input variables. Furthermore, Yang and Wang^51^ analyzed the application of EGRs to the urban canopy by applying Monte Carlo simulations, Mazzeo et al.^24^ investigated the effect of EGRs on the temperature at the rooftop level using artificial neural networks, and Suter et al.^30^ developed an equation for calculating the air temperature variation in the surface layer (i.e., from roof height to 280 m) depending on GRs. Although such studies provide useful information, such as roof surface temperature^24,51^ and domain-averaged evaporation rate^30^, such data can hardly be used by urban planners to evaluate the UHI mitigation potential at the pedestrian level of adaptation plans. The model developed in this study is based on both regression models and microclimate simulations conducted with ENVI-met, a tool based on the laws of thermodynamics and fluid dynamics, which can simulate the interactions between air, surfaces, and vegetation in an urban context^36^. Therefore, the output reliability depends on the accuracy of both the ENVI-met software and the regression models. Previous research has enlisted ENVI-met as a primary tool capable of accurately predicting air temperature under different meteorological conditions^48^ as well as the effect of BIVTs on microclimate^20,21^. In addition, the regression models have been found to be significant and explains 0.61 and 0.68 of the data variances for EGRs and GFs, respectively, and for both, the residuals are normal and homoscedastic. On the other hand, the LW model is found to be significant, and its residuals are homoscedastic, but its adjusted R^2^ equals 0.50, and its residuals are nonnormal. Nevertheless, by including the effect of the different urban arrangements as intercepts, the accuracy of the LW model greatly improves (i.e., adjusted R^2^ = 0.63), and its residuals are normal (i.e., p-val = 0.12). Therefore, as far as the LWs are concerned, the reliability of the model greatly depends on the urban arrangement. However, adding the urban arrangement effect might hinder the generalizability of the current model.

The developed EGR regression model revealed a direct correlation between both the LAI and COP and the median air temperature. Our results are in accordance with those of Suter et al.^30^ regarding coverage. With respect to the LAI, our findings are in agreement with those of Jamel et al.^52^, even though they observed that after a certain LAI threshold, the temperature reduction reaches a plateau. Furthermore, we found that increasing the COP is more effective for mitigation than increasing the LAI. This finding was also confirmed by Iaria and Susca^35^. In contrast, Fig. 3 also shows an indirect correlation between BH and air temperature mitigation, meaning that the effectiveness of EGRs in mitigating excessively high air temperature at the pedestrian level decreases when the height of the rooftops where EGRs are installed increases. This result is in accordance with previous literature (e.g.,^37^) and is explained by the greater distance of the mitigation source from the target. Furthermore, as in^35^, we found that a BH equal to approximately 40 m can be considered a threshold above which the application of EGRs has a negligible effect on air temperature reduction at the pedestrian level^38,53^.

As far as the GF and LW models are concerned, BH was found to be a negligible input parameter in comparison with LAI and COP and was therefore removed from the models. This finding might indicate that proximity to the target is as important—or even more—as the greened area increases in mitigating urban air overheating at the desired height. Indeed, the mitigation effect of GFs and LWs at the pedestrian level is greater when the source of evapotranspiration is closer to the target (i.e., street level). Nevertheless, the installation of GF and LW-based mitigation scenarios might be beneficial for reducing the urban temperature at the rooftop level. This result should be further investigated in future studies. In Figs. 4 and 5, both the GF and LW models, respectively, predict a direct relationship between both the LAI and COP and the mitigation of excessively high air temperature. When applying GFs, low values of LAI (e.g., LAI = 1.5) lead to a negligible median reduction or even an increase in temperature, while high values (e.g., LAI = 5) lead to a median reduction of approximately 0.1 °C. Moreover, when GFs are deployed, an increase in LAI is more effective than an increase in COP in mitigating excessively high air temperature. In contrast, in the application of LWs, the effect of an increase in COP on temperature mitigation (ranging from approximately − 0.04 °C with COP = 25% to approximately − 0.12 °C with COP = 100%) is significantly greater than that of an increase in LAI (ranging from approximately − 0.07 °C with LAI = 1.5 to approximately − 0.09 °C with COP = 100%). It is worth noting that there is an exiguous difference in the mitigation potential of LW-based adaptation scenarios. Specifically, LAI 3 scenarios seem to be slightly better performing than LAI 5. Such a result might be justified by the fact that very dense vegetation might trap some heat and prevent its dissipation^11^.

**Figure 6:**
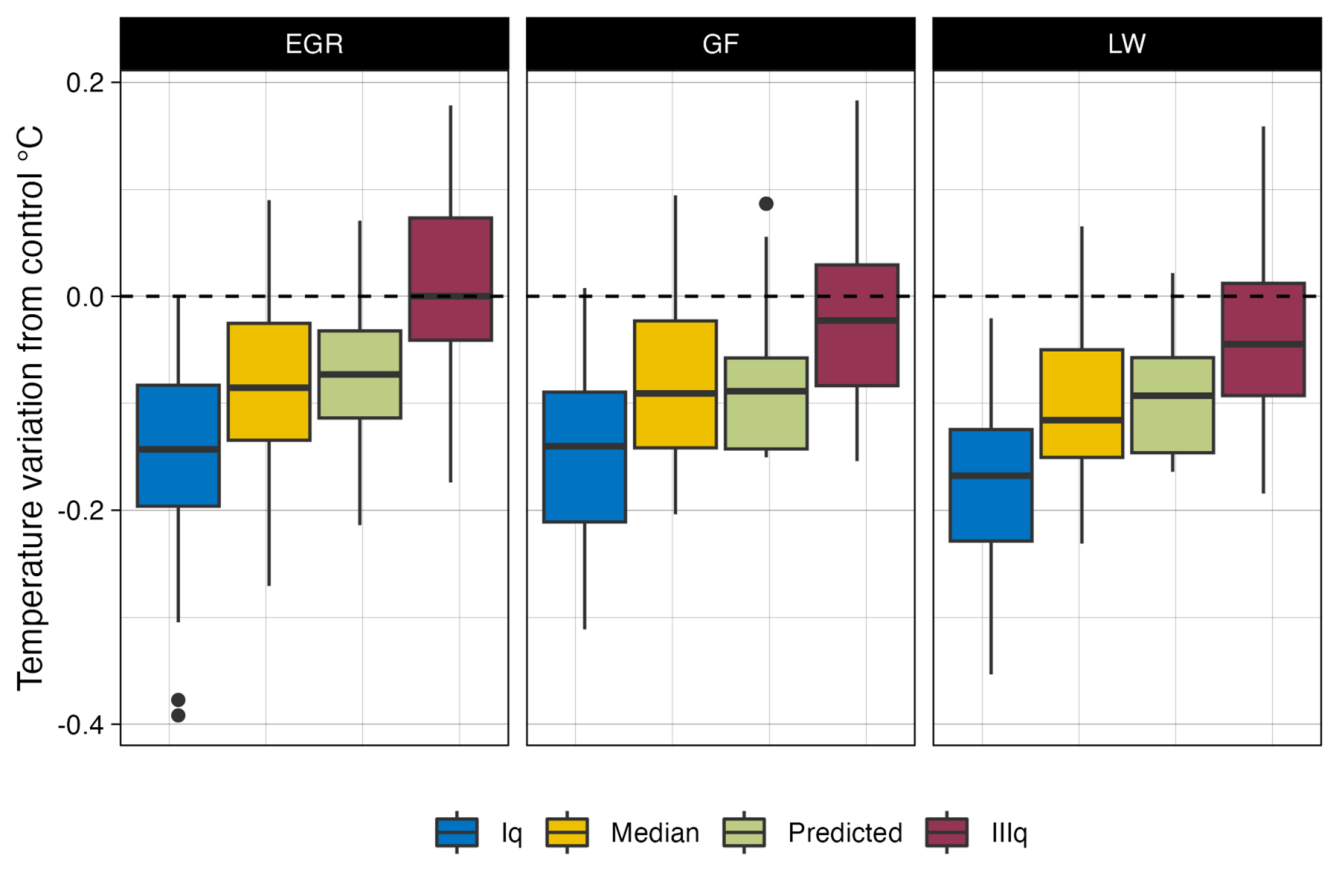
Boxplots of the 25th, 50th, and 75th percentiles of the spatial observed and predicted temperature mitigation values.

As far as limitations and shortcomings are concerned, one limitation of the proposed model resides in its applicability to the Mediterranean climate only, specifically, the Csa climate zone. Nonetheless, the same methodology might be applied to other climate zones. Furthermore, climatic variables such as wind direction and speed, which have an impact on mitigation^38^, were kept constant in the study areas by using typical summer days to force climatic conditions. Future research might improve the index with changing climatic variables to obtain insights into mitigation performance under changing conditions. Additionally, the model assumes that GRs and GWs are fully irrigated at any time. The performance of BIVTs depends on irrigation and, in particular, water availability for plants^12,54^; therefore, under real conditions, water availability is a crucial factor. Moreover, the vegetation layer of the BIVTs is composed of standard, well-investigated plants such as *Sedum sediforme, Nephrolepis exalata,* and *Hedera helix* for EGRs, LWs, and GFs, respectively. We neglected the use of different local species because they were outside the scope of the study, but as transpiration is dependent on plant traits, differences might occur when other species are implemented in BIVTs. By applying a single BIVT, most of the mitigation values range from 0 to − 0.2 °C. Such values seem to be negligible; nevertheless, mitigation is not evenly distributed within these areas. The effect of BIVTs tends to be distributed near buildings or in the upwind direction. Specifically, in 25% of the urban areas, the temperature mitigation is significantly greater than the median value (Fig. 6), and punctual mitigation values can reach − 0.83 °C.

Nevertheless, the proposed models might also indicate the potential ineffectiveness of BIVT-based adaptation plans. Such result is equally important since it might entail the implementation of more synergic solution sets^19^.

Eventually, the proposed methodology focuses on the urban overheating mitigation potential of BIVT-based adaptation plans excluding other mitigation measures that might be part of UHI adaptation solution sets such as an increase in urban albedo^19^. Furthermore, the present study omits the evaluation of the potential synergy of photovoltaic panels and GRs installation that can underpin the positive effects of both technologies^19,55^. Both different mitigation solution sets and photovoltaic panels might be considered in a further study.

## Conclusions

The present study reports on the development of three indices to predict the mitigation potential of single BIVT-based scenarios—specifically, EGRs, GFs, and LWs—to eradicate UHIs in urban areas in the Csa climate zone characterized by an urban density ranging from 0.3 to 0.4. The developed indices can be applied to built areas by varying the parameters that most influence the UHI mitigation potential of BIVT-based scenarios: BH, COP, and LAI.

Such indices are useful since they can facilitate the work of planners and city council technicians who want to assess beforehand the effectiveness of BIVT-based UHI adaptation plans. The developed indices can be used to forecast and compare the effects of adaptation BIVT-based plans using easily available data to overcome the limits of other methods (i.e.,^36^), and their outputs remain within an acceptable range of error.

Furthermore, the indices are novel since no studies have been found in the published literature providing such a tool, and unlike other research that focuses on providing punctual temperature variation values, the current study provides spatial median variations in urban temperature, which can more reliably support urban planners in designing UHI adaptation plans. The novelty of the proposed indices also resides in the use of a methodology based both on the development of 281 ENVI-met models and simulations and on linear regression models. The proposed methodology can be used for developing indices for other climate zones. The precision of the model greatly relies on that of the ENVI-met tool; therefore, in the future, when the ENVI-met model will be further improved, the developed indices, combined with other simulations, might decrease their error. However, limitations exist. For instance, the model might be further enriched with other parameters, such as plant watering data, or models can be developed for single urban arrangements. However, although the precision of the model would probably benefit from such implementations, the implementation of more parameters would likely increase the complexity of the indices, and the focus on single urban arrangements would make the indices less applicable; altogether, such improvements might hinder urban planners from their use.

To conclude, the developed indices can greatly contribute to easing the ability of planners to eradicate urban overheating, and the proposed methodology can pave the way for the development of other indices capable of assessing, with different degrees of error, the effectiveness of UHI adaptation plans.

## Supplementary Information

Supplementary Information.

## Data Availability

The datasets necessary for preparing ENVI-met simulations are available in the^35^ supplementary materials. All ENVI-met-related files, extracted temperature layers from simulation results, R scripts, and data generated for computing models are available upon request to Jacopo Iaria, email: jacopo.iaria@unibo.it.

## References

[1] Landsberg, H. E. *The Urban Climate* (Academic Press, 1981).

[2] Zhang, P., Imhoff, M. L., Wolfe, R. E. & Bounoua, L. Characterizing urban heat islands of global settlements using MODIS and nighttime lights products. *Can. J. Remote. Sens.* **36**, 185–196 (2010).

[3] Huang, W. T. K. *et al.* Economic valuation of temperature-related mortality attributed to urban heat islands in European cities. *Nat. Commun.* **14**, 7438 (2023). 37978178 10.1038/s41467-023-43135-z PMC10656443

[4] Santamouris, M., Cartalis, C., Synnefa, A. & Kolokotsa, D. On the impact of urban heat island and global warming on the power demand and electricity consumption of buildings—A review. *Energy Build.* **98**, 119–124 (2015).

[5] Santamouris, M. On the energy impact of urban heat island and global warming on buildings. *Energy Build.* **82**, 100–113 (2014).

[6] Li, H. *et al.* Interaction between urban heat island and urban pollution island during summer in Berlin. *Sci. Total Environ.* **636**, 818–828 (2018). 29727848 10.1016/j.scitotenv.2018.04.254

[7] Chessa, C. & Susca, T. Development of an LCA characterization factor to account UHI local effect on terrestrial ecosystems damage category: Evaluation of European Bombus and Onthophagus genera heat-stress mortality. *Sci. Total Environ.* **897**, 165183 (2023). 37385499 10.1016/j.scitotenv.2023.165183

[8] Wong, N. H., Tan, C. L., Kolokotsa, D. D. & Takebayashi, H. Greenery as a mitigation and adaptation strategy to urban heat. *Nat. Rev. Earth Environ.* **2**, 166–181 (2021).

[9] Edmondson, J. L., Stott, I., Davies, Z. G., Gaston, K. J. & Leake, J. R. Soil surface temperatures reveal moderation of the urban heat island effect by trees and shrubs. *Sci. Rep.* **6**, 33708 (2016). 27641002 10.1038/srep33708 PMC5027384

[10] Susca, T., Zanghirella, F. & Del Fatto, V. Building integrated vegetation effect on micro-climate conditions for urban heat island adaptation Lesson learned from Turin and Rome case studies. *Energy Build.* **295**, 113233 (2023).

[11] Susca, T., Zanghirella, F., Colasuonno, L. & Del Fatto, V. Effect of green wall installation on urban heat island and building energy use: A climate-informed systematic literature review. *Renew. Sustain. Energy Rev.* **159**, 112100 (2022).

[12] Susca, T. Green roofs to reduce building energy use? A review on key structural factors of green roofs and their effects on urban climate. *Build. Environ.* **162**, 106273 (2019).

[13] Dunnett, N. & Kingsbury, N. *Planting Green Roofs and Living Walls* Vol. VII (Timber Press, 2004).

[14] Environmental Protection Agency. Reducing Urban Heat Islands: Compendium of Strategies. Green Roofs. (2008).

[15] Castleton, H. F., Stovin, V., Beck, S. B. M. & Davison, J. B. Green roofs; building energy savings and the potential for retrofit. *Energy Build.* **42**, 1582–1591 (2010).

[16] Manso, M. & Castro-Gomes, J. Green wall systems: A review of their characteristics. *Renew. Sustain. Energy Rev.* **41**, 863–871 (2015).

[17] London City Council. London Plan 2011. *London City Hall* https://www.london.gov.uk//what-we-do/planning/london-plan/past-versions-and-alterations-london-plan/london-plan-2011 (2011).

[18] Irga, P. J. *et al.* The distribution of green walls and green roofs throughout Australia: Do policy instruments influence the frequency of projects?. *Urban For. Urban Green.* **24**, 164–174 (2017).

[19] Zhao, Y. *et al.* Beating urban heat: Multimeasure-centric solution sets and a complementary framework for decision-making. *Renew. Sustain. Energy Rev.* **186**, 113668 (2023).

[20] Liu, J., Zhang, L., Zhang, Q., Zhang, G. & Teng, J. Predicting the surface urban heat island intensity of future urban green space development using a multi-scenario simulation. *Sustain. Cities Soc.* **66**, 102698 (2021).

[21] Liu, Z. *et al.* Modeling microclimatic effects of trees and green roofs/façades in ENVI-met: Sensitivity tests and proposed model library. *Build. Environ.* **244**, 110759 (2023).

[22] Ouyang, W. *et al.* Evaluating the thermal-radiative performance of ENVI-met model for green infrastructure typologies: Experience from a subtropical climate. *Build. Environ.* **207**, 108427 (2022).

[23] Balany, F., Ng, A. W., Muttil, N., Muthukumaran, S. & Wong, M. S. Green infrastructure as an urban heat island mitigation strategy—a review. *Water* **12**, 3577 (2020).

[24] Mazzeo, D., Matera, N., Peri, G. & Scaccianoce, G. Forecasting green roofs’ potential in improving building thermal performance and mitigating urban heat island in the Mediterranean area: An artificial intelligence-based approach. *Appl. Therm. Eng.* **222**, 119879 (2023).

[25] Sinsel, T., Simon, H., Broadbent, A. M., Bruse, M. & Heusinger, J. Modeling the outdoor cooling impact of highly radiative “super cool” materials applied on roofs. *Urban Clim.* **38**, 100898 (2021).

[26] Onishi, A., Cao, X., Ito, T., Shi, F. & Imura, H. Evaluating the potential for urban heat-island mitigation by greening parking lots. *Urban For. Urban Green.* **9**, 323–332 (2010).

[27] Mohammad, P., Goswami, A., Chauhan, S. & Nayak, S. Machine learning algorithm based prediction of land use land cover and land surface temperature changes to characterize the surface urban heat island phenomena over Ahmedabad city, India. *Urban Clim.* **42**, 101116 (2022).

[28] Shen, C. *et al.* Prediction of the future urban heat island intensity and distribution based on landscape composition and configuration: A case study in Hangzhou. *Sustain. Cities Soc.* **83**, 103992 (2022).

[29] Zawadzka, J. E., Harris, J. A. & Corstanje, R. Assessment of heat mitigation capacity of urban greenspaces with the use of InVEST urban cooling model, verified with day-time land surface temperature data. *Landsc. Urban Plan.* **214**, 104163 (2021).

[30] Suter, I., Maksimović, Č & van Reeuwijk, M. A neighbourhood-scale estimate for the cooling potential of green roofs. *Urban Clim.* **20**, 33–45 (2017).

[31] Stewart, I. D. & Oke, T. R. Local climate zones for urban temperature studies. *Bull. Am. Meteorol. Soc.* **93**, 1879–1900 (2012).

[32] Diffenbaugh, N. S., Pal, J. S., Giorgi, F. & Gao, X. Heat stress intensification in the Mediterranean climate change hotspot. *Geophys. Res. Lett.*10.1029/2007GL030000 (2007).

[33] Katavoutas, G. & Founda, D. Intensification of thermal risk in Mediterranean climates: Evidence from the comparison of rational and simple indices. *Int. J. Biometeorol.* **63**, 1251–1264 (2019). 31201549 10.1007/s00484-019-01742-w

[34] Kottek, M., Grieser, J., Beck, C., Rudolf, B. & Rubel, F. World Map of the Köppen-Geiger climate classification updated. *metz* **15**, 259–263 (2006).

[35] Iaria, J. & Susca, T. Analytic Hierarchy Processes (AHP) evaluation of green roof- and green wall- based UHI mitigation strategies via ENVI-met simulations. *Urban Clim.* **46**, 101293 (2022).

[36] High-Resolution 3D Modeling of Urban Microclimate with ENVI-met Software. *ENVI-met* https://www.envi-met.com/.

[37] Ng, E., Chen, L., Wang, Y. & Yuan, C. A study on the cooling effects of greening in a high-density city: An experience from Hong Kong. *Build. Environ.* **47**, 256–271 (2012).

[38] Jin, C., Bai, X., Luo, T. & Zou, M. Effects of green roofs’ variations on the regional thermal environment using measurements and simulations in Chongqing, China. *Urban For. Urban Green.* **29**, 223–237 (2018).

[39] CTI Comitato Termotecnico Italiano. https://www.cti2000.it/.

[40] Morakinyo, T. E., Lai, A., Lau, K.K.-L. & Ng, E. Thermal benefits of vertical greening in a high-density city: Case study of Hong Kong. *Urban For. Urban Green.* **37**, 42–55 (2019).

[41] Morakinyo, T. E., Dahanayake, K. W. D. K. C., Ng, E. & Chow, C. L. Temperature and cooling demand reduction by green-roof types in different climates and urban densities: A co-simulation parametric study. *Energy Build.* **145**, 226–237 (2017).

[42] Support Data Analysis and Visualization Tools from Envi-met | Tutorials, Modeling Guides & More. *ENVI-met* https://envi-met.com/envi-met-support-area/.

[43] ENVI-met Support Center - Best number of extra calculation hour for initialization. http://www.envi-hq.com/viewtopic.php?f=3&t=3999&sid=09da056bff63dd7a6159fe516151fd89.

[44] R: The R Project for Statistical Computing. https://www.r-project.org/.

[45] Zeileis, A. & Hothorn, T. Diagnostic Checking in Regression Relationships. *R News* vol. 2 (2002).

[46] GitHub, Inc., Footer navigation & mfrasco. mfrasco/Metrics. (2024).

[47] Tiedemann, F. erocoar/gghalves. (2024).

[48] Tsoka, S., Tsikaloudaki, A. & Theodosiou, T. Analyzing the ENVI-met microclimate model’s performance and assessing cool materials and urban vegetation applications—A review. *Sustain. Cities Soc.* **43**, 55–76 (2018).

[49] Alexandri, E. & Jones, P. Temperature decreases in an urban canyon due to green walls and green roofs in diverse climates. *Build. Environ.* **43**, 480–493 (2008).

[50] Djedjig, R., Bozonnet, E. & Belarbi, R. Modeling green wall interactions with street canyons for building energy simulation in urban context. *Urban Clim.* **16**, 75–85 (2016).

[51] Yang, J. & Wang, Z.-H. Physical parameterization and sensitivity of urban hydrological models: Application to green roof systems. *Build. Environ.* **75**, 250–263 (2014).

[52] Jamei, E. *et al.* Investigating the cooling effect of a green roof in Melbourne. *Build. Environ.* **246**, 110965 (2023).

[53] Chen, H., Ooka, R., Huang, H. & Tsuchiya, T. Study on mitigation measures for outdoor thermal environment on present urban blocks in Tokyo using coupled simulation. *Build. Environ.* **44**, 2290–2299 (2009).

[54] Vaezizadeh, F., Rashidisharifabad, S. & Afhami, R. Investigating the cooling effect of living walls in the sunken courtyards of traditional houses in Yazd. *Eur. J. Sustain. Dev.* **5**, 27–27 (2016).

[55] Manso, M., Teotónio, I., Silva, C. M. & Cruz, C. O. Green roof and green wall benefits and costs: A review of the quantitative evidence. *Renew. Sustain. Energy Rev.* **135**, 110111 (2021).

