# Supplementary material for: Development of predictive indices for evaluating the UHI adaptation potential of green roof- and wall-based scenarios in the Mediterranean climate
Source: Sci Rep. 2024 Sep 17;14:21675. doi: 10.1038/s41598-024-67567-9 (PMC11408658; doi:10.1038/s41598-024-67567-9)
Supplement: Supplementary file 1 — Supplementary Information. [file 41598_2024_67567_MOESM1_ESM.docx]

Supplementary Material

Tab.S.1: All developed scenarios

| **Control scenario Name** | **City** | **ENVI-met Version** | **Corresponding mitigation scenario name** | **Tech** | **COP (%)** | **BH (m)** | **LAI** |
| --- | --- | --- | --- | --- | --- | --- | --- |
| C_H05_BARI_v5.1 | Bari | 5.1 | EGR_H05_COP25_BA | EGR | 25 | 5 | 5 |
|  |  |  | EGR_H05_COP50_BA | EGR | 50 | 5 | 5 |
|  |  |  | EGR_H05_COP75_BA | EGR | 75 | 5 | 5 |
|  |  |  | EGR_H05_LAI1_BA | EGR | 100 | 5 | 1.5 |
|  |  |  | GF_H05_COP25_BA | GF | 25 | 5 | 5 |
|  |  |  | GF_H05_COP50_BA | GF | 50 | 5 | 5 |
|  |  |  | GF_H05_COP75_BA | GF | 75 | 5 | 5 |
|  |  |  | GF_H05_LAI1_BA | GF | 100 | 5 | 1.5 |
|  |  |  | LW_H05_COP25_BA | LW | 25 | 5 | 5 |
|  |  |  | LW_H05_COP50_BA | LW | 50 | 5 | 5 |
|  |  |  | LW_H05_COP75_BA | LW | 75 | 5 | 5 |
|  |  |  | LW_H05_LAI1_BA | LW | 100 | 5 | 1.5 |
| C_H10_BARI_v5.1 | Bari | 5.1 | EGR_H10_COP25_BA | EGR | 25 | 10 | 5 |
|  |  |  | EGR_H10_COP50_BA | EGR | 50 | 10 | 5 |
|  |  |  | EGR_H10_LAI1_BA | EGR | 100 | 10 | 1.5 |
|  |  |  | GF_H10_COP25_BA | GF | 25 | 10 | 5 |
|  |  |  | GF_H10_COP50_BA | GF | 50 | 10 | 5 |
|  |  |  | GF_H10_COP75_BA | GF | 75 | 10 | 5 |
|  |  |  | LW_H10_COP25_BA | LW | 25 | 10 | 5 |
|  |  |  | LW_H10_COP50_BA | LW | 50 | 10 | 5 |
|  |  |  | LW_H10_COP75_BA | LW | 75 | 10 | 5 |
|  |  |  | LW_H10_LAI1_BA | LW | 100 | 10 | 1.5 |
| C_H20_BARI_v5.1 | Bari | 5.1 | EGR_H20_COP25_BA | EGR | 25 | 20 | 5 |
|  |  |  | EGR_H20_COP50_BA | EGR | 50 | 20 | 5 |
|  |  |  | EGR_H20_COP75_BA | EGR | 75 | 20 | 5 |
|  |  |  | EGR_H20_LAI1_BA | EGR | 100 | 20 | 1.5 |
|  |  |  | GF_H20_COP25_BA | GF | 25 | 20 | 5 |
|  |  |  | GF_H20_COP50_BA | GF | 50 | 20 | 5 |
|  |  |  | GF_H20_COP75_BA | GF | 75 | 20 | 5 |
|  |  |  | GF_H20_LAI1_BA | GF | 100 | 20 | 1.5 |
|  |  |  | LW_H20_COP25_BA | LW | 25 | 20 | 5 |
|  |  |  | LW_H20_COP50_BA | LW | 50 | 20 | 5 |
|  |  |  | LW_H20_COP75_BA | LW | 75 | 20 | 5 |
|  |  |  | LW_H20_LAI1_BA | LW | 100 | 20 | 1.5 |
| C_H30_BARI_v5.1 | Bari | 5.1 | EGR_H30_LAI1_BA | EGR | 100 | 30 | 1.5 |
| base_bari_dati_palese_v5.1 | Bari | 5.1 | EGR_COP25_LAI1_BA | EGR | 25 | 20 | 1.5 |
|  |  |  | EGR_COP50_LAI1_BA | EGR | 50 | 20 | 1.5 |
|  |  |  | EGR_COP75_LAI1_BA | EGR | 75 | 20 | 1.5 |
|  |  |  | GF_COP25_LAI3_BA | GF | 25 | 20 | 3 |
|  |  |  | GF_COP50_LAI3_BA | GF | 50 | 20 | 3 |
|  |  |  | GF_COP75_LAI3_BA | GF | 75 | 20 | 3 |
|  |  |  | GF_COP25_LAI1_BA | GF | 25 | 20 | 1.5 |
|  |  |  | GF_COP50_LAI1_BA | GF | 50 | 20 | 1.5 |
|  |  |  | GF_COP75_LAI1_BA | GF | 75 | 20 | 1.5 |
|  |  |  | LW_COP25_LAI3_BA | LW | 25 | 20 | 3 |
|  |  |  | LW_COP50_LAI3_BA | LW | 50 | 20 | 3 |
|  |  |  | LW_COP75_LAI3_BA | LW | 75 | 20 | 3 |
|  |  |  | LW_COP25_LAI1_BA | LW | 25 | 20 | 1.5 |
|  |  |  | LW_COP50_LAI1_BA | LW | 50 | 20 | 1.5 |
|  |  |  | LW_COP75_LAI1_BA | LW | 75 | 20 | 1.5 |
| H05C_FI_v5.1 | Florence | 5.1 | EGR_H05_COP25_FI | EGR | 25 | 5 | 5 |
|  |  |  | EGR_H05_COP50_FI | EGR | 50 | 5 | 5 |
|  |  |  | EGR_H05_COP75_FI | EGR | 75 | 5 | 5 |
|  |  |  | EGR_H05_LAI1_FI | EGR | 100 | 5 | 1.5 |
|  |  |  | GF_H05_COP25_FI | GF | 25 | 5 | 5 |
|  |  |  | GF_H05_COP50_FI | GF | 50 | 5 | 5 |
|  |  |  | GF_H05_COP75_FI | GF | 75 | 5 | 5 |
|  |  |  | GF_H05_LAI1_FI | GF | 100 | 5 | 1.5 |
|  |  |  | LW_H05_COP25_FI | LW | 25 | 5 | 5 |
|  |  |  | LW_H05_COP50_FI | LW | 50 | 5 | 5 |
|  |  |  | LW_H05_COP75_FI | LW | 75 | 5 | 5 |
|  |  |  | LW_H05_LAI1_FI | LW | 100 | 5 | 1.5 |
| H10C_FI_v5.1 | Florence | 5.1 | EGR_H10_COP25_FI | EGR | 25 | 10 | 5 |
|  |  |  | EGR_H10_COP50_FI | EGR | 50 | 10 | 5 |
|  |  |  | EGR_H10_COP75_FI | EGR | 75 | 10 | 5 |
|  |  |  | EGR_H10_LAI1_FI | EGR | 100 | 10 | 1.5 |
|  |  |  | GF_H10_COP25_FI | GF | 25 | 10 | 5 |
|  |  |  | GF_H10_COP50_FI | GF | 50 | 10 | 5 |
|  |  |  | GF_H10_COP75_FI | GF | 75 | 10 | 5 |
|  |  |  | GF_H10_LAI1_FI | GF | 100 | 10 | 1.5 |
|  |  |  | LW_H10_COP25_FI | LW | 25 | 10 | 5 |
|  |  |  | LW_H10_COP50_FI | LW | 50 | 10 | 5 |
|  |  |  | LW_H10_COP75_FI | LW | 75 | 10 | 5 |
|  |  |  | LW_H10_LAI1_FI | LW | 100 | 10 | 1.5 |
| H20C_FI_v5.1 | Florence | 5.1 | EGR_H20_COP25_FI | EGR | 25 | 20 | 5 |
|  |  |  | EGR_H20_COP50_FI | EGR | 50 | 20 | 5 |
|  |  |  | EGR_H20_COP75_FI | EGR | 75 | 20 | 5 |
|  |  |  | EGR_H20_LAI1_FI | EGR | 100 | 20 | 1.5 |
|  |  |  | GF_H20_COP25_FI | GF | 25 | 20 | 5 |
|  |  |  | GF_H20_COP50_FI | GF | 50 | 20 | 5 |
|  |  |  | GF_H20_COP75_FI | GF | 75 | 20 | 5 |
|  |  |  | GF_H20_LAI1_FI | GF | 100 | 20 | 1.5 |
|  |  |  | LW_H20_COP25_FI | LW | 25 | 20 | 5 |
|  |  |  | LW_H20_COP50_FI | LW | 50 | 20 | 5 |
|  |  |  | LW_H20_COP75_FI | LW | 75 | 20 | 5 |
|  |  |  | LW_H20_LAI1_FI | LW | 100 | 20 | 1.5 |
| base_firenze_datiPeretola_v5.1 | Florence | 5.1 | EGR_COP25_LAI1_FI | EGR | 25 | 15 | 1.5 |
|  |  |  | EGR_COP50_LAI1_FI | EGR | 50 | 15 | 1.5 |
|  |  |  | EGR_COP75_LAI1_FI | EGR | 75 | 15 | 1.5 |
|  |  |  | GF_COP25_LAI1_FI | GF | 25 | 15 | 1.5 |
|  |  |  | GF_COP50_LAI1_FI | GF | 50 | 15 | 1.5 |
|  |  |  | GF_COP75_LAI1_FI | GF | 75 | 15 | 1.5 |
|  |  |  | GF_COP25_LAI3_FI | GF | 25 | 15 | 3 |
|  |  |  | GF_COP50_LAI3_FI | GF | 50 | 15 | 3 |
|  |  |  | GF_COP75_LAI3_FI | GF | 75 | 15 | 3 |
|  |  |  | LW_COP25_LAI1_FI | LW | 25 | 15 | 1.5 |
|  |  |  | LW_COP50_LAI1_FI | LW | 50 | 15 | 1.5 |
|  |  |  | LW_COP75_LAI1_FI | LW | 75 | 15 | 1.5 |
|  |  |  | LW_COP25_LAI3_FI | LW | 25 | 15 | 3 |
|  |  |  | LW_COP50_LAI3_FI | LW | 50 | 15 | 3 |
|  |  |  | LW_COP75_LAI3_FI | LW | 75 | 15 | 3 |
| C_H05_RM_v5.1 | Rome | 5.1 | EGR_H05_COP25_RM | EGR | 25 | 5 | 5 |
|  |  |  | EGR_H05_COP50_RM | EGR | 50 | 5 | 5 |
|  |  |  | EGR_H05_COP75_RM | EGR | 75 | 5 | 5 |
|  |  |  | EGR_H05_LAI1_RM | EGR | 100 | 5 | 1.5 |
|  |  |  | EGR_H05_LAI5_RM | EGR | 100 | 5 | 5 |
|  |  |  | GF_H05_COP25_RM | GF | 25 | 5 | 5 |
|  |  |  | GF_H05_COP50_RM | GF | 50 | 5 | 5 |
|  |  |  | GF_H05_COP75_RM | GF | 75 | 5 | 5 |
|  |  |  | GF_H05_LAI1_RM | GF | 100 | 5 | 1.5 |
|  |  |  | LW_H05_COP25_RM | LW | 25 | 5 | 5 |
|  |  |  | LW_H05_COP50_RM | LW | 50 | 5 | 5 |
|  |  |  | LW_H05_COP75_RM | LW | 75 | 5 | 5 |
|  |  |  | LW_H05_LAI1_RM | LW | 100 | 5 | 1.5 |
| C_H10_RM_v5.1 | Rome | 5.1 | EGR_H10_COP25_RM | EGR | 25 | 10 | 5 |
|  |  |  | EGR_H10_COP50_RM | EGR | 50 | 10 | 5 |
|  |  |  | EGR_H10_COP75_RM | EGR | 75 | 10 | 5 |
|  |  |  | EGR_H10_LAI1_RM | EGR | 100 | 10 | 1.5 |
|  |  |  | EGR_H10_LAI5_RM | EGR | 100 | 10 | 5 |
|  |  |  | GF_H10_COP25_RM | GF | 25 | 10 | 5 |
|  |  |  | GF_H10_COP50_RM | GF | 50 | 10 | 5 |
|  |  |  | GF_H10_COP75_RM | GF | 75 | 10 | 5 |
|  |  |  | GF_H10_LAI1_RM | GF | 100 | 10 | 1.5 |
|  |  |  | LW_H10_COP25_RM | LW | 25 | 10 | 5 |
|  |  |  | LW_H10_COP50_RM | LW | 50 | 10 | 5 |
|  |  |  | LW_H10_COP75_RM | LW | 75 | 10 | 5 |
|  |  |  | LW_H10_LAI1_RM | LW | 100 | 10 | 1.5 |
| C_H20_RM_v5.1 | Rome | 5.1 | EGR_H20_COP25_RM | EGR | 25 | 20 | 5 |
|  |  |  | EGR_H20_COP50_RM | EGR | 50 | 20 | 5 |
|  |  |  | EGR_H20_COP75_RM | EGR | 75 | 20 | 5 |
|  |  |  | GF_H20_COP25_RM | GF | 25 | 20 | 5 |
|  |  |  | GF_H20_COP50_RM | GF | 50 | 20 | 5 |
|  |  |  | GF_H20_COP75_RM | GF | 75 | 20 | 5 |
|  |  |  | GF_H20_LAI1_RM | GF | 100 | 20 | 1.5 |
|  |  |  | LW_H20_COP25_RM | LW | 25 | 20 | 5 |
|  |  |  | LW_H20_COP50_RM | LW | 50 | 20 | 5 |
|  |  |  | LW_H20_COP75_RM | LW | 75 | 20 | 5 |
|  |  |  | LW_H20_LAI1_RM | LW | 100 | 20 | 1.5 |
| lanciani_base_v5.1 | Rome | 5.1 | EGR_COP25_LAI1_RM | EGR | 25 | 20 | 1.5 |
|  |  |  | EGR_COP25_LAI5_RM | EGR | 25 | 20 | 5 |
|  |  |  | EGR_COP50_LAI1_RM | EGR | 50 | 20 | 1.5 |
|  |  |  | EGR_COP50_LAI5_RM | EGR | 50 | 20 | 5 |
|  |  |  | EGR_COP75_LAI1_RM | EGR | 75 | 20 | 1.5 |
|  |  |  | EGR_COP75_LAI5_RM | EGR | 75 | 20 | 5 |
|  |  |  | EGR_H20_LAI1_RM | EGR | 100 | 20 | 1.5 |
|  |  |  | GF_COP25_LAI1_RM | GF | 25 | 20 | 1.5 |
|  |  |  | GF_COP50_LAI1_RM | GF | 50 | 20 | 1.5 |
|  |  |  | GF_COP75_LAI1_RM | GF | 75 | 20 | 1.5 |
|  |  |  | LW_COP25_LAI1_RM | LW | 25 | 20 | 1.5 |
|  |  |  | LW_COP50_LAI1_RM | LW | 50 | 20 | 1.5 |
|  |  |  | LW_COP75_LAI1_RM | LW | 75 | 20 | 1.5 |
| C_H05_BARI | Bari | 4.4.6 | GR_H05E_BARI | EGR | 100 | 5 | 5 |
|  |  |  | GF_H05_BARI | GF | 100 | 5 | 5 |
|  |  |  | LW_H05_BARI | LW | 100 | 5 | 5 |
| C_H10_BARI | Bari | 4.4.6 | GR_H10E_BARI | EGR | 100 | 10 | 5 |
|  |  |  | GF_H10_BARI | GF | 100 | 10 | 5 |
|  |  |  | LW_H10_BARI | LW | 100 | 10 | 5 |
| C_H20_BARI | Bari | 4.4.6 | GR_H20E_BARI | EGR | 100 | 20 | 5 |
|  |  |  | GF_H20_BARI | GF | 100 | 20 | 5 |
|  |  |  | LW_H20_BARI | LW | 100 | 20 | 5 |
| C_H30_BARI | Bari | 4.4.6 | GR_H30E_BARI | EGR | 100 | 30 | 5 |
|  |  |  | GF_H30_BARI | GF | 100 | 30 | 5 |
|  |  |  | LW_H30_BARI | LW | 100 | 30 | 5 |
| C_H40_BARI | Bari | 4.4.6 | GR_H40E_BARI | EGR | 100 | 40 | 5 |
|  |  |  | GF_H40_BARI | GF | 100 | 40 | 5 |
|  |  |  | LW_H40_BARI | LW | 100 | 40 | 5 |
| base_bari_dati_palese | Bari | 4.4.6 | GR_COP25E_BARI | EGR | 25 | 20 | 5 |
|  |  |  | GR_COP50E_BARI | EGR | 50 | 20 | 5 |
|  |  |  | GR_COP75E_BARI | EGR | 75 | 20 | 5 |
|  |  |  | GRE_COP100_BARI | EGR | 100 | 20 | 5 |
|  |  |  | GRE_LAI1_BARI | EGR | 100 | 20 | 1.5 |
|  |  |  | GRE_LAI3_BARI | EGR | 100 | 20 | 3 |
|  |  |  | GF_COP_100_BARI | GF | 100 | 20 | 5 |
|  |  |  | GF_COP25_BARI | GF | 25 | 20 | 5 |
|  |  |  | GF_COP50_BARI | GF | 50 | 20 | 5 |
|  |  |  | GF_COP75_BARI | GF | 75 | 20 | 5 |
|  |  |  | GF_LAI1_BARI | GF | 100 | 20 | 1.5 |
|  |  |  | GF_LAI3_BARI | GF | 100 | 20 | 3 |
|  |  |  | LW_COP100_BARI | LW | 100 | 20 | 5 |
|  |  |  | LW_COP25_BARI | LW | 25 | 20 | 5 |
|  |  |  | LW_COP50_BARI | LW | 50 | 20 | 5 |
|  |  |  | LW_COP75_BARI | LW | 75 | 20 | 5 |
|  |  |  | LW_LAI1_BARI | LW | 100 | 20 | 1.5 |
|  |  |  | LW_LAI3_BARI | LW | 100 | 20 | 3 |
| H05C_FI | Florence | 4.4.6 | GR_H05E_FI | EGR | 100 | 5 | 5 |
|  |  |  | GF_H05_FI | GF | 100 | 5 | 5 |
|  |  |  | LW_H05_FI | LW | 100 | 5 | 5 |
| H10C_FI_v5.1 | Florence | 4.4.6 | GR_H10E_FI | EGR | 100 | 10 | 5 |
|  |  |  | GF_H10_FI | GF | 100 | 10 | 5 |
|  |  |  | LW_H10_FI | LW | 100 | 10 | 5 |
| H20C_FI | Florence | 4.4.6 | GR_H20E_FI | EGR | 100 | 20 | 5 |
|  |  |  | GF_H20_FI | GF | 100 | 20 | 5 |
|  |  |  | LW_H20_FI | LW | 100 | 20 | 5 |
| H30C_FI | Florence | 4.4.6 | GR_H30E_FI | EGR | 100 | 30 | 5 |
|  |  |  | GF_H30_FI | GF | 100 | 30 | 5 |
|  |  |  | LW_H30_FI | LW | 100 | 30 | 5 |
| H40C_FI | Florence | 4.4.6 | GR_H40E_FI | EGR | 100 | 40 | 5 |
|  |  |  | GF_H40_FI | GF | 100 | 40 | 5 |
|  |  |  | LW_H40_FI | LW | 100 | 40 | 5 |
| base_firenze_datiPeretola | Florence | 4.4.6 | GR_COP100E_FI | EGR | 100 | 15 | 5 |
|  |  |  | GR_COP25E_FI | EGR | 25 | 15 | 5 |
|  |  |  | GR_COP50E_FI | EGR | 50 | 15 | 5 |
|  |  |  | GR_COP75E_FI | EGR | 75 | 15 | 5 |
|  |  |  | GR_LAI1E_FI | EGR | 100 | 15 | 1.5 |
|  |  |  | GR_LAI3E_FI | EGR | 100 | 15 | 3 |
|  |  |  | GF_COP100_FI | GF | 100 | 15 | 5 |
|  |  |  | GF_COP25_FI | GF | 25 | 15 | 5 |
|  |  |  | GF_COP50_FI | GF | 50 | 15 | 5 |
|  |  |  | GF_COP75_FI | GF | 75 | 15 | 5 |
|  |  |  | GF_LAI1_FI | GF | 100 | 15 | 1.5 |
|  |  |  | GF_LAI3_FI | GF | 100 | 15 | 3 |
|  |  |  | LW_COP100_FI | LW | 100 | 15 | 5 |
|  |  |  | LW_COP25_FI | LW | 25 | 15 | 5 |
|  |  |  | LW_COP50_FI | LW | 50 | 15 | 5 |
|  |  |  | LW_COP75_FI | LW | 75 | 15 | 5 |
|  |  |  | LW_LAI1_FI | LW | 100 | 15 | 1.5 |
|  |  |  | LW_LAI3_FI | LW | 100 | 15 | 3 |
| C_H05_RM | Rome | 4.4.6 | EGR_H05_RM | EGR | 100 | 5 | 3 |
|  |  |  | GF_H05_RM | GF | 100 | 5 | 5 |
|  |  |  | LW_H05_RM | LW | 100 | 5 | 5 |
| C_H10_RM | Rome | 4.4.6 | EGR_H10_RM | EGR | 100 | 10 | 3 |
|  |  |  | GF_H10_RM | GF | 100 | 10 | 5 |
|  |  |  | LW_H10_RM | LW | 100 | 10 | 5 |
| C_H20_RM | Rome | 4.4.6 | EGR_H20_RM | EGR | 100 | 20 | 5 |
|  |  |  | GF_H20_RM | GF | 100 | 20 | 5 |
|  |  |  | LW_H20_RM | LW | 100 | 20 | 5 |
| C_H30_RM | Rome | 4.4.6 | EGR_H30_RM | EGR | 100 | 30 | 3 |
|  |  |  | GF_H30_RM | GF | 100 | 30 | 5 |
|  |  |  | LW_H30_RM | LW | 100 | 30 | 5 |
| C_H40_RM | Rome | 4.4.6 | EGR_H40_RM | EGR | 100 | 40 | 3 |
|  |  |  | GF_H40_RM | GF | 100 | 40 | 5 |
|  |  |  | LW_H40_RM | LW | 100 | 40 | 5 |
| lanciani_base | Rome | 4.4.6 | EGR_COP100_RM | EGR | 100 | 20 | 5 |
|  |  |  | EGR_COP25_RM | EGR | 25 | 20 | 3 |
|  |  |  | EGR_COP50_RM | EGR | 50 | 20 | 3 |
|  |  |  | EGR_COP75_RM | EGR | 75 | 20 | 3 |
|  |  |  | EGR_LAI1_RM | EGR | 100 | 20 | 1.5 |
|  |  |  | EGR_LAI3_RM | EGR | 100 | 20 | 3 |
|  |  |  | GF_COP100_RM | GF | 100 | 20 | 5 |
|  |  |  | GF_COP25_RM | GF | 25 | 20 | 5 |
|  |  |  | GF_COP50_RM | GF | 50 | 20 | 5 |
|  |  |  | GF_COP75_RM | GF | 75 | 20 | 5 |
|  |  |  | GF_LAI1_RM | GF | 100 | 20 | 1.5 |
|  |  |  | GF_LAI3_RM | GF | 100 | 20 | 3 |
|  |  |  | LW_COP100_RM | LW | 100 | 20 | 5 |
|  |  |  | LW_COP25_RM | LW | 25 | 20 | 5 |
|  |  |  | LW_COP50_RM | LW | 50 | 20 | 5 |
|  |  |  | LW_COP75_RM | LW | 75 | 20 | 5 |
|  |  |  | LW_LAI1_RM | LW | 100 | 20 | 1.5 |
|  |  |  | LW_LAI3_RM | LW | 100 | 20 | 3 |

| 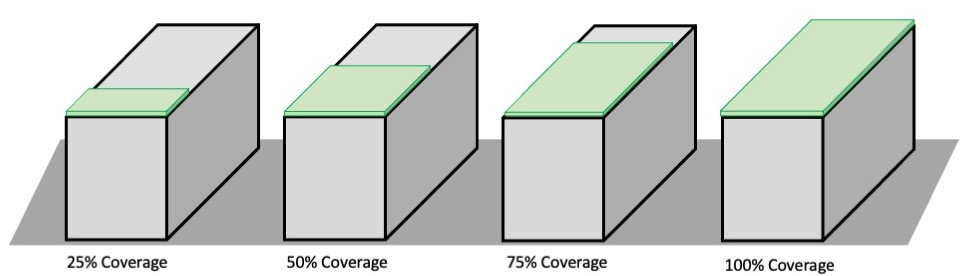 |
| --- |
| Supplementary figure.1: scenarios of 25, 50, 75, and 100% coverage of EGR |

| 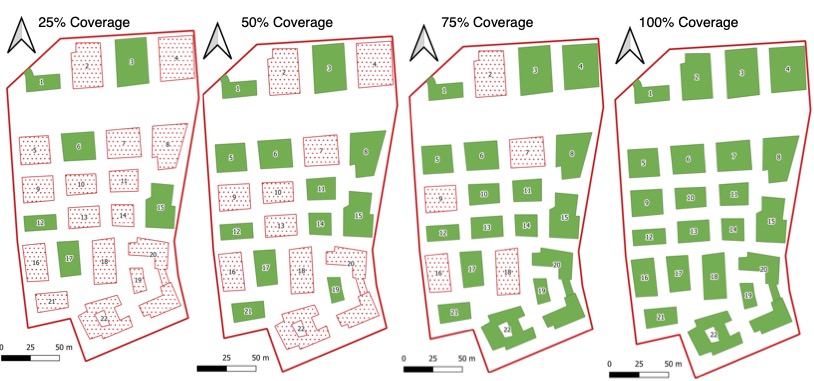 |
| --- |
| Supplementary figure.2: buildings covered by GWs in 25, 50, 75, and 100% coverage scenarios, respectively, in Rome via Lanciani |

| 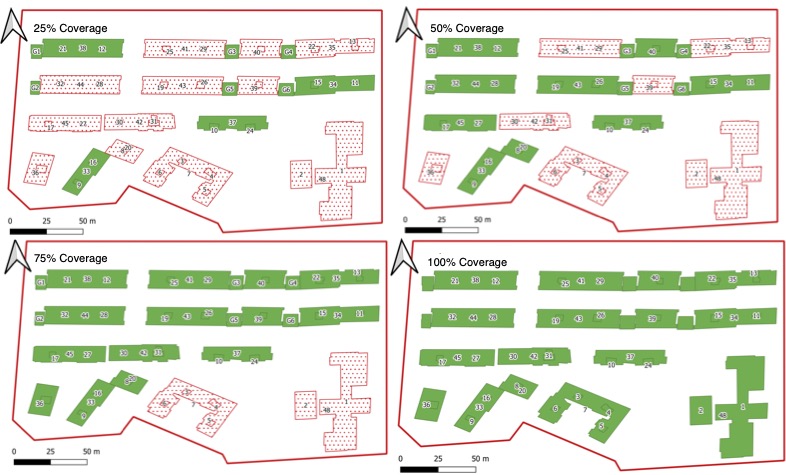 |
| --- |
| Supplementary figure.3: buildings covered by GWs in 25, 50, 75, and 100% coverage scenarios, respectively, in Bari, Viale Kennedy |

| 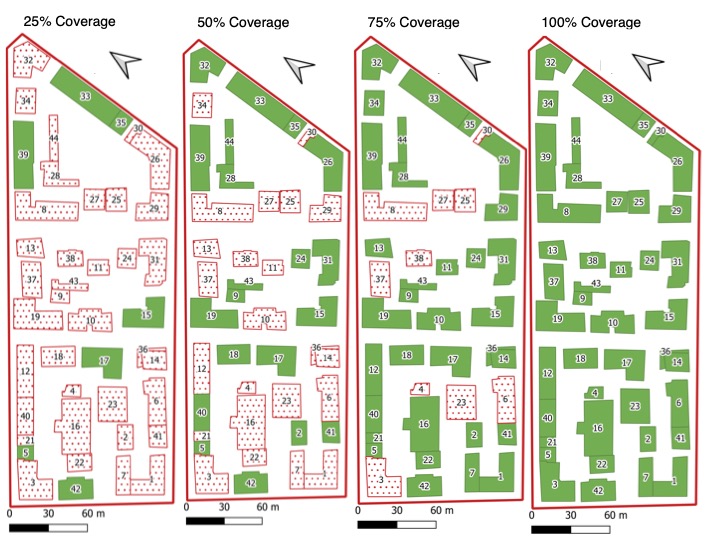 |
| --- |
| Supplementary figure.4: buildings covered by GWs in 25, 50, 75, and 100% coverage scenarios, respectively, in Florence Gavinana |
